# Supplementary material for: AMPK deficiency in smooth muscles causes persistent pulmonary hypertension of the new-born and premature death
Source: Nat Commun. 2022 Aug 26;13:5034. doi: 10.1038/s41467-022-32568-7 (PMC9418192; doi:10.1038/s41467-022-32568-7)
Supplement: Supplementary file 1 — Supplementary information [file 41467_2022_32568_MOESM1_ESM.pdf]

Supplementary Information

AMPK deficiency in smooth muscles causes persistent pulmonary hypertension of the new-born and premature death

Javier Moral-Sanz BSc, PhD[1], Sophronia A. Lewis BSc, PhD [1], Sandy MacMillan BSc, PhD [1], Marco Meloni BSc, PhD [2], Heather McClafferty, Benoit Viollet BSc, PhD [4,5,6], Marc Foretz BSc, PhD [4,5,6], Jorge del-Pozo BSc, MSc, PhD [3], A. Mark Evans BSc, PhD [1]

[1]Centre for Discovery Brain Sciences and Cardiovascular Science, College of Medicine and Veterinary Medicine, Hugh Robson Building, University of Edinburgh, Edinburgh, EH8 9XD, UK. [2]Centre for Cardiovascular Science, Queen's Medical Research Institute, University of Edinburgh, Edinburgh, EH16 4TJ, UK. [3]R(D)SVS, University of Edinburgh Easter Bush Campus, EH2 9RG, UK. [4]Institut Cochin, INSERM U1016, [5]CNRS UMR 8104 and [6]Université Paris Descartes, Sorbonne Paris cité, Paris, France.

Supplementary Methods

Western Blot Liver, kidney, ventricle and atria were lysed by sonication 3 x 15 sec in lysis buffer (50mM tris, 50 mM NaF, 5 mM sodium pyrophosphate, 1mM EDTA, 1 mM EGTA, PH7.4 at 4° C, 1% triton X, 1 mM DTT, 5μg/ml soyabean trypsin inhibitor, 0.1 mM PMSF, 1 mM Benzamidine), centrifuged at 6500 rpm, 30 sec x 4. 33μg, 55μg (aorta only) or 100 μg of each lysate was run on gels. AMPK subunit protein expression was analyzed using precast 4-12% BisTris gels (Invitrogen, product code WG1403BOX) in MOPS running buffer (Invitrogen, NP000102). Molecular weight ladder was SeeBlue plus 2 (Invitrogen product code LC5925). Proteins were transferred to nitrocellulose membranes using an Xcell II blot module and probed with antibodies against AMPK subunits. AMPK-α1 (ab3759, cell signalling, 1:1000 dilution) and AMPK-α2 (ab105028, cell signalling, 1:1000 dilution) antibodies were Abcam. GAPDH antibody (2118S, 1:10,000 dilution) was from cell signalling.

TASK-1 expressing HEK293 cells were generated as follows. Full length cDNAs encoding human TASK-1 (hTASK-1) channels, a kind gift from Dr. S. A. N. Goldstein (Department of Pediatrics and Institute for Molecular Pediatric Sciences, Pritzker School of Medicine, University of Chicago), were originally subcloned into the mammalian expression vectors pRAT and pMAX(+) via Bgl II/Sal I and Xba I/Xma I restriction site combinations respectively. To generate HEK293 cell lines stably expressing wild-type hTASK-1, cells were transfected with either pRAT/hTASK-1 constructs using the PolyFect transfection reagent (Qiagen, Hybaid Ltd, Teddington, UK) according to manufacturer's instructions. Stable HEK293 cell lines were achieved by antibiotic selection with G-418 (1mg/ml, Gibco-BRL, Paisley, UK) added to the medium 3 days after transfection. Selection was applied for 4 weeks (media changed every 4-5 days), after which time individual colonies were picked and seeded in T25 flasks and allowed to reach confluence. They were then transferred to T75 flasks for further culture and electrophysiological screening. Cells were harvested from culture flasks by trypsinization and plated onto coverslips 24-48h before use in electrophysiological studies. Transfection of hTASK-1 channels was considered successful if the currents elicited by the whole-cell voltage ramp protocol (see below) were: a) significantly (> 4-fold) larger than untransfected HEK293 cell K^+ currents; b) reliably described by the Goldman Hodgkin and Katz (GHK) equation and; c) activated

by pH 8.4 and inhibited by pH 6.4 (and the pH-sensitive currents demonstrated GHK rectification). In addition, hTASK-1 was insensitive to ruthenium red, while hTASK3 was sensitive as previously described (Czirjak and Enydei Mol Pharm. 2003; 63: 646-652; DOI: https://doi.org/10.1124/mol.63.3.646). Whole-cell patch-clamp recordings were recorded from HEK293 cells stably expressing wild-type hTASK-1. Coverslip fragments with attached cells were transferred to a continuously perfused recording chamber (perfusion rate 3-5 ml/min, volume ca 200µl) mounted on the stage of an inverted microscope. Cells were perfused with a solution containing (in mM): 135 NaCl, 5 KCl, 1.2 $MgCl_2$, 5 HEPES, 2.5 $CaCl_2$, 10 D-glucose (pH 7.4 with KOH). Patch electrodes (resistance 4-7MΩ) were filled with intracellular solution consisting of (in mM): 10 NaCl, 117 KCl, 2 $MgCl_2$, 11 HEPES, 11 EGTA, 1 $CaCl_2$, 2 Na_2ATP (pH 7.2 with KOH). All chemicals were obtained from Sigma-Aldrich (Poole, UK). Outward K^+ currents were recorded at 37°C (unless otherwise stated) using standard ramp protocols; cells were voltage-clamped at a holding potential of –70mV, then stepped to -100mV and a voltage ramp (500ms duration) immediately applied from -100 mV to +60mV. Data were acquired and digitized via a Digidata 1322A in combination with an Axopatch 200B amplifier and Clampex 9 software (Molecular Devices, Foster City, CA). Currents were sampled at 2kHz and low-pass filtered at 1kHz. Offline data analysis was conducted using Clampfit 9 software (Molecular Devices, Foster City, CA.). Differences were considered significant when $P<0.05$. All values stated are as mean±SEM.

a

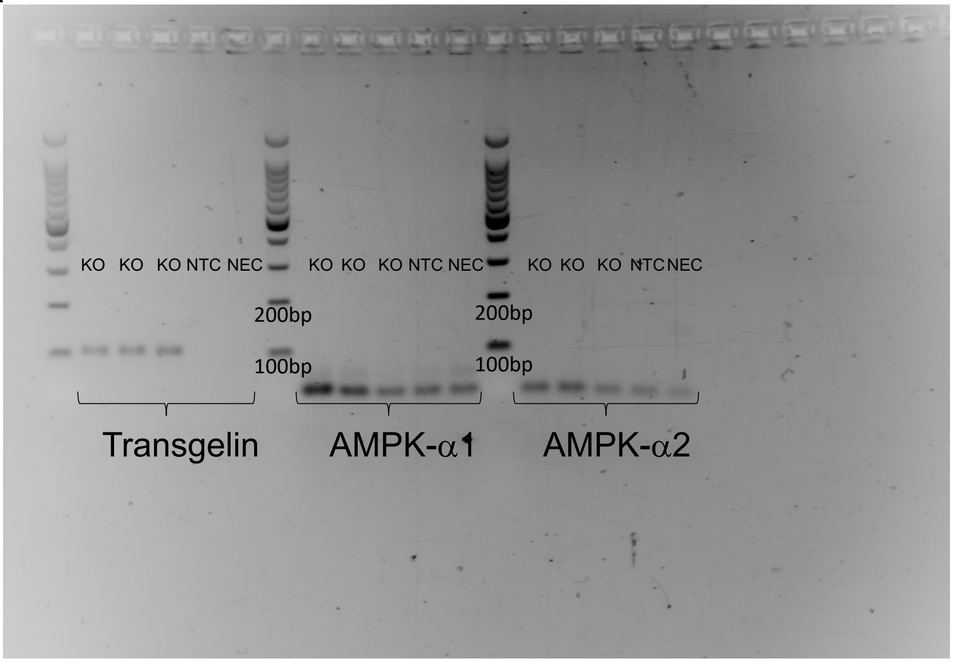

b

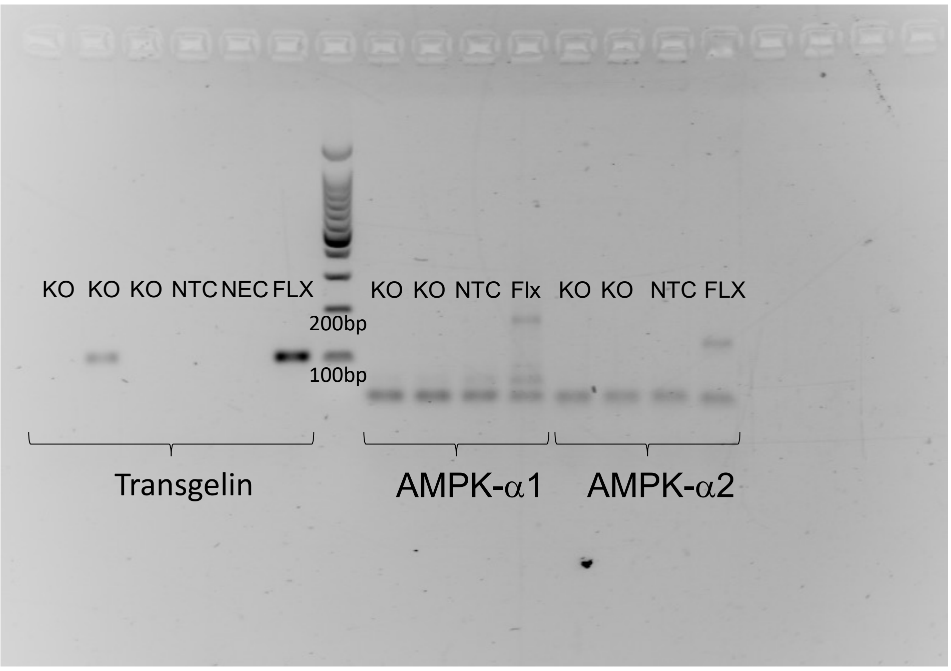

Supplementary Fig 1. Raw gels for end-point PCR on laser microdissected pulmonary arterial smooth muscles from AMPK-α1/α2 knockouts. a and b show raw gels for single cell quantitative RT-PCR amplicons for transgelin, AMPK-α1 and AMPK-α2 from AMPK-α1/α2 knockout and AMPK-α1/α2 floxed (FLX) mice. Transgelin expression and samples from floxed mice were used as positive controls. NEC = no enzyme control (no reverse transcriptase added). NTC = no template control (cell aspirant but no cell reverse transcriptase added). For (a) n = 1 sample was run in triplicate. For (b) n = 4 different samples were run. Samples were taken from two different AMPK-α1/α2 knockout mice.

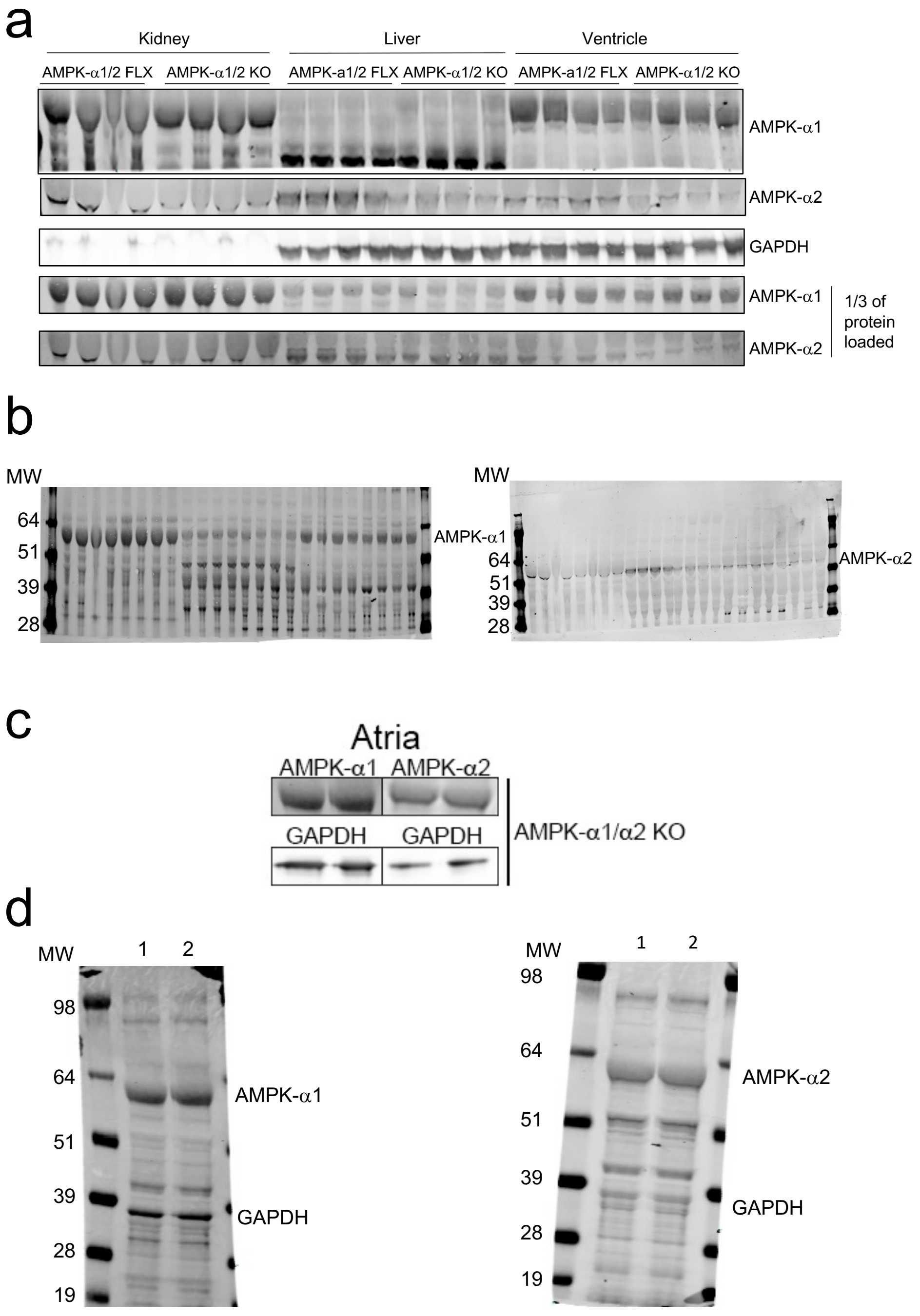

Supplementary Figure 2. Western blot reveals no loss of AMPK-α1 or AMPK-α2 expression in cardiac ventricles following AMPK-α1/α2 deletion by transgelin-Cre. a, Exemplar western blots for AMPK-α1 and AMPK-α2 protein levels in kidney, liver and left ventricle of the heart for AMPK-α1/α2 Floxed (AMPK-α1/α2 FLX) and AMPK-α1/α2 knockout (AMPK-α1/α2 KO) mice, relative to loading control (GAPDH). b, raw gels for western blot images shown in (a). c, Exemplar western blots for AMPK-α1 and AMPK-α2 protein levels in the atria of AMPK-α1/α2 knockout (KO) mice, relative to loading control (GAPDH). d, raw gels for western blot images shown in (b). For ventricles, liver and kidneys samples from n = 4 AMPK-α1/α2 KO mice were run in triplicate. For atria samples from n = 2 AMPK-α1/α2 KO mice were run in triplicate.

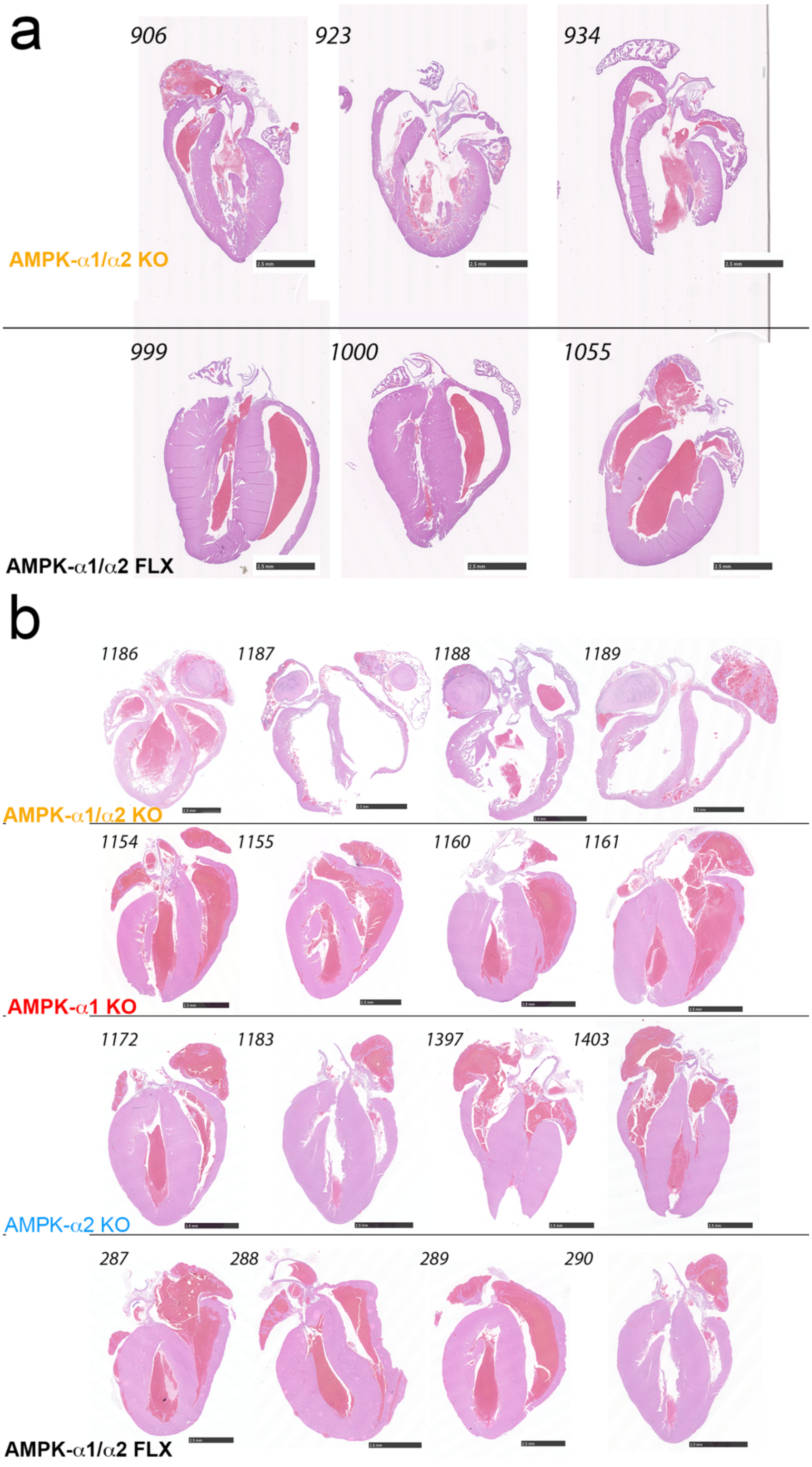

Supplementary Figure 3. Remodelling of the heart in terminal samples from AMPK-α1/α2 knockouts. Sub-gross images of heart slices from (a) non-terminal and (b) terminal samples stained with Hematoxylin-Eosin for: AMPK-α1/α2 knockouts (AMPK-α1/α2 KO, n = 4 mice); AMPK-α1 knockout (AMPK-α1 KO; n = 4 mice); AMPK-α2 knockout (AMPK-α2 KO; n = 4 mice); AMPK-α1/α2 floxed (AMPK-α1/α2 FLX; n = 4 mice). Scale bars 2.5mm.

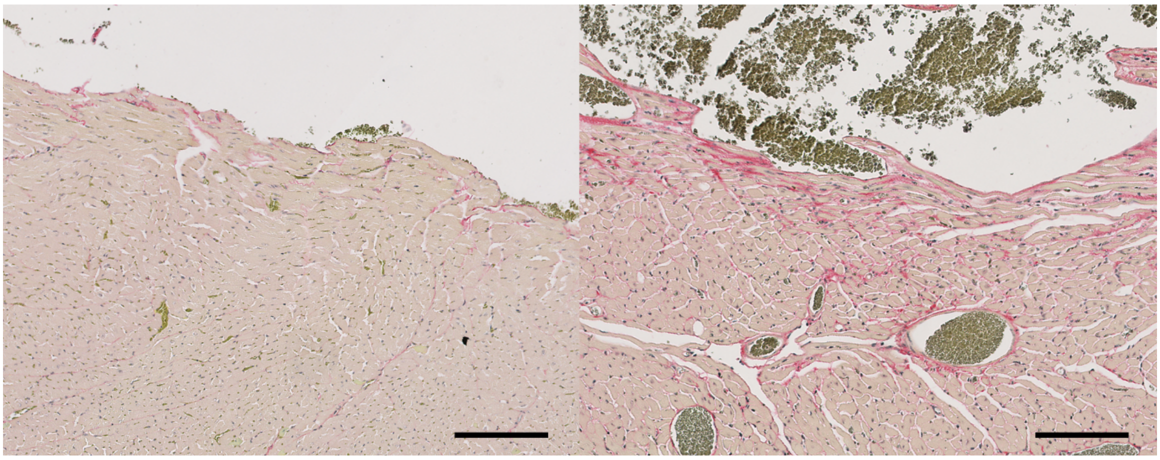

Supplementary Figure 4. AMPK-α1/α2 deletion precipitates fibrosis of the left ventricle. Exemplar images of the left ventricle from age-matched AMPK-α1/α2 FLX (left; n = 4 mice) and AMPK-α1/α2 knockout (right; n = 4 mice) mice shows relative Picro-Sirius Red staining (red; erythrocytes are stained green (not analysed), counterstain is pale yellow. Scale bars 100 μm.

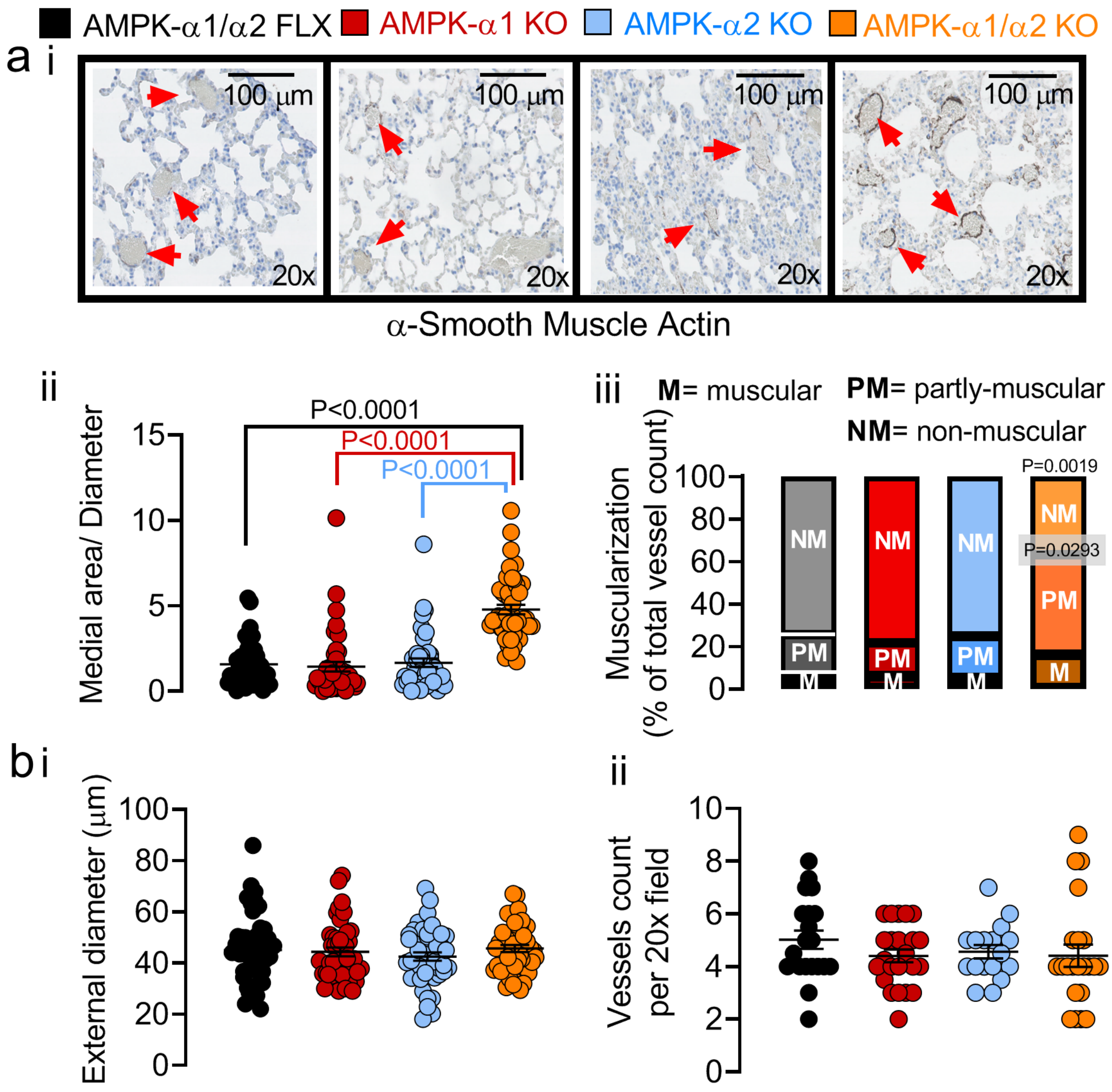

Supplementary Figure 5. Analysis of pulmonary arterial remodelling triggered by AMPK-α1/α2 deletion by total vessel counts rather than by mouse. ai, Representative images of lung slices from terminal samples stained for α-smooth muscle actin, together with scatter plots of (ii) medial area corrected by diameter and (iii) degree of muscularization for all pulmonary arteries analysed for knockout mice (AMPK-α1/α2 KO; n = 125 arteries, n = 7 fields/mouse, 4 mice) after death at 7-10 weeks, and age-matched AMPK-α1/α2 floxed mice (AMPK-α1/α2 Flx; n = 141 arteries, 7 fields/mouse, 4 mice), AMPK-α1 KOs (n = 122 arteries, n = 7 fields per mouse, n = 4 mice) and AMPK-α2 KOs (n=136 arteries, 7 fields/mouse, 4 mice). b, Scatter plots show the mean ± SEM for the (i) external diameter for, 122, 136 for AMPK-α1/α2 KO (n = 125 arteries counted from n = 4 mice), AMPK- α1/α2 floxed (n = 141 arteries counted from n = 4 mice), AMPK-α1 KO (n = 122 arteries counted from n = 4 mice) and AMPK-α KO (n = 136 arteries counted from n = 4 mice) and (ii) number of vessels found per 20x field (n = 28 fields per genotype) for the analysis shown in b(i). For clarity, only mean values are presented in panel aiii. The rest of the panels are expressed as mean± SEM. Statistical significance was assessed by one-way ANOVA (aii, bi-ii) or a two-way ANOVA (aiii) with Dunn's correction for multiple comparisons.

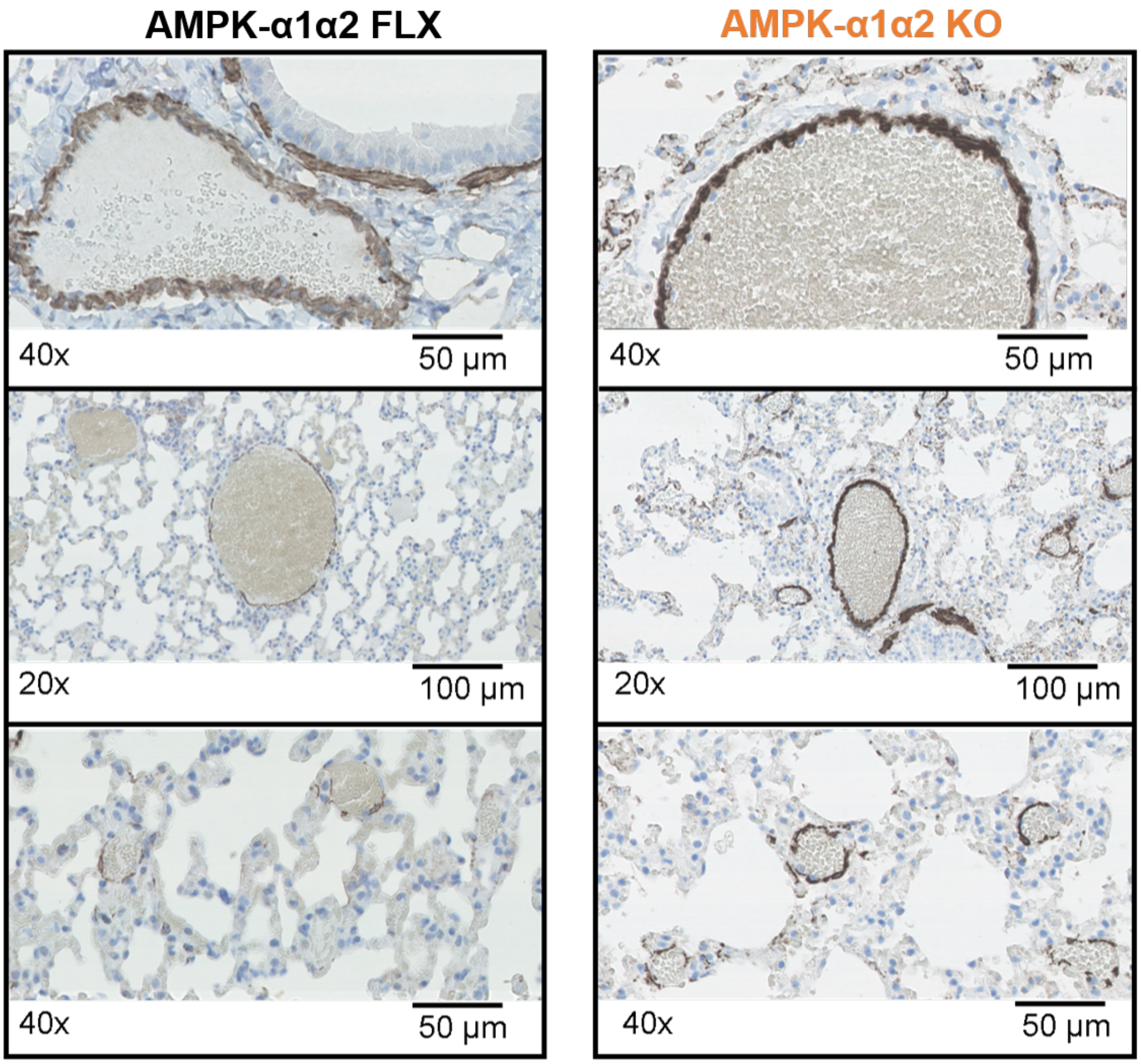

Supplementary Figure 6. Remodelling of pulmonary arterial media in AMPK-α1/α2 knockouts. Representative high-resolution images of pulmonary arteries in terminal lung slices stained for α-smooth muscle actin from replicates taken from n = 4 AMPK-α1/α2 Floxed (AMPK-α1/α2 FLX; left) and n = 4 AMPK-α1/α2 knockout (AMPK-α1/α2 KO; right) mice.

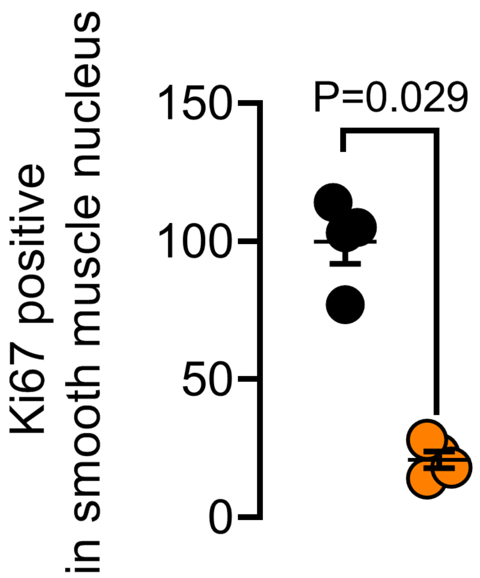

Supplementary Figure 7. Reduced Ki67 positive nuclei in pulmonary arterial myocytes of AMPK-α1/α2 knockouts after birth. Scatter plot shows the mean ± SEM for Ki67 labelled nuclei in smooth muscle cells of pulmonary arteries in-situ within lung slices from AMPK-α1/α2 FLX (black, n = 4 mice) and AMPK-α1/α2 KO (orange, n=4 mice). Statistical significance was assessed by two-sided unpaired Mann-Whitney's test.

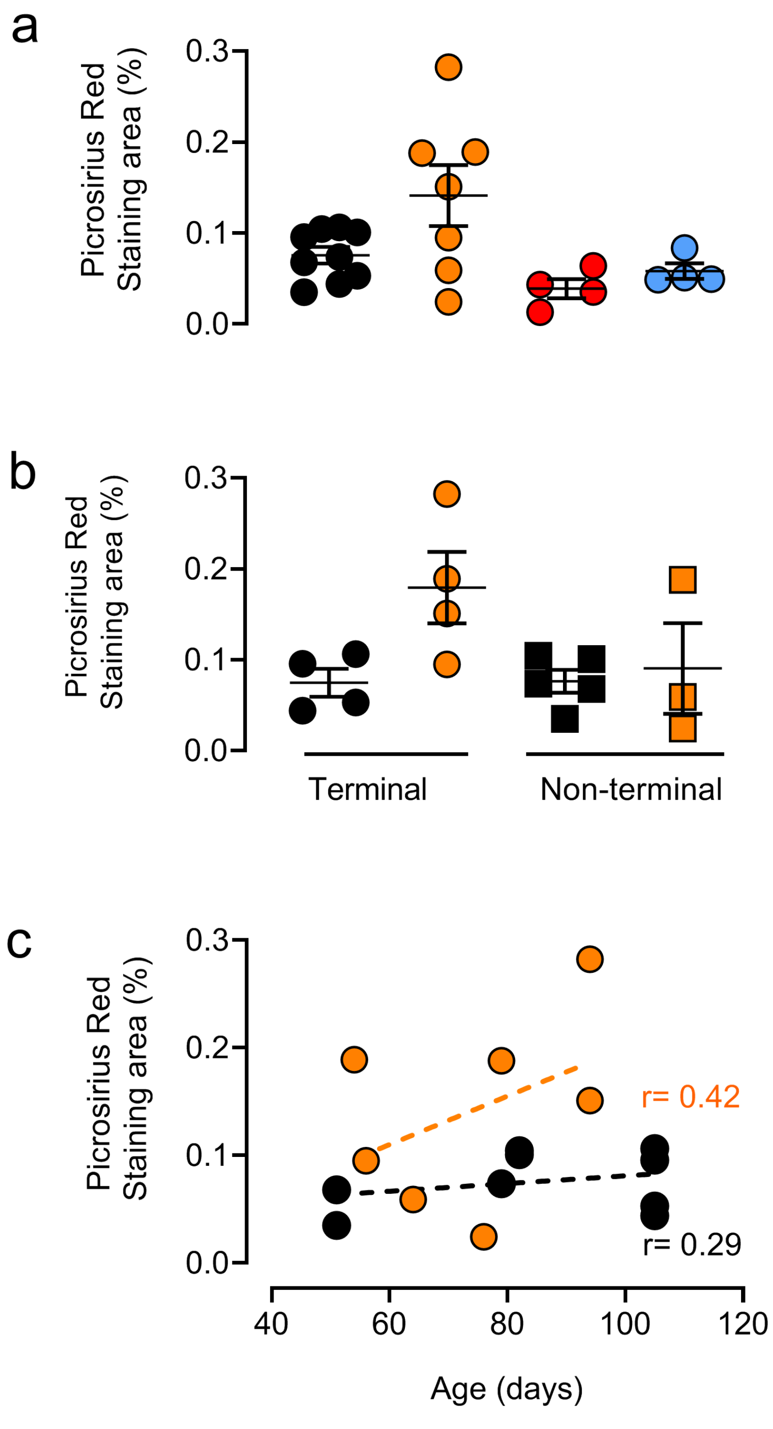

Supplementary Figure 8. Parenchymal fibrosis is not triggered by AMPK-α1/α2 deletion. a, Scatter plots show the mean ± SEM for analysis of Picro-Sirius Red staining. AMPK-α1/α2 FLX (black, n = 8 mice) and AMPK-α1/α2 KO (orange, n = 7 mice), AMPK-α1 KO (red, n = 4 mice), AMPK-α2 KO (blue, n = 4 mice). b, as in A but for comparison of terminal and non-terminal samples from AMPK-α1/α2 KO (n=4 mice and n=3 mice, respectively) and AMPK-α1/α2 FLX (n=4 mice). c, linear regression analysis of Picro-Sirius Red staining versus age. Statistical significance was assessed by Kruskall Wallis test with Dunn's correction for multiple comparisons (a), two- sided unpaired Mann-Whitney's test (b) and two-sided Pearson correlation coefficients (c).

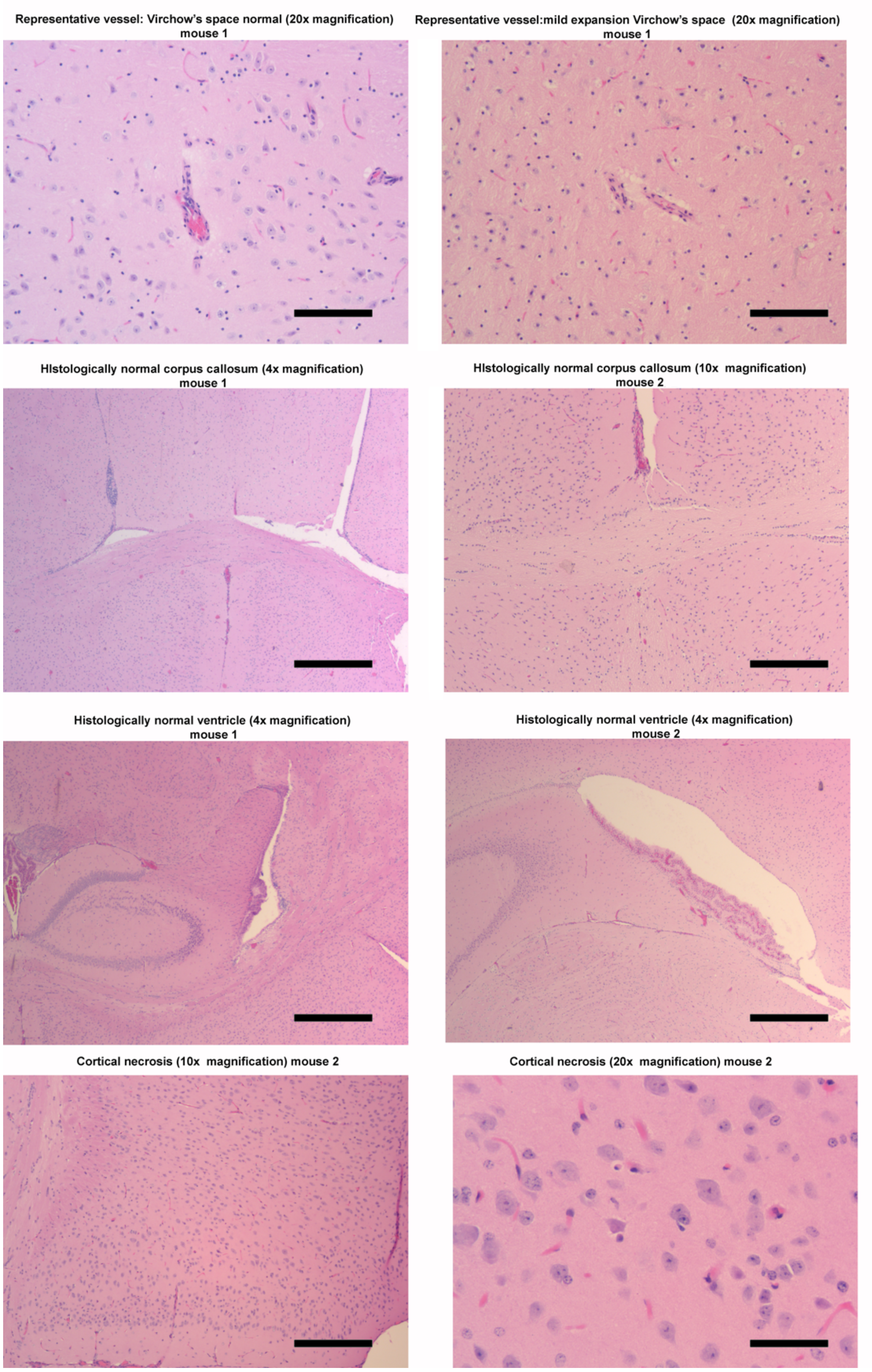

Supplementary Figure 9. Histology on terminal AMPK-α1/α2 knockouts indicates no lesion of the brain. Panels, from top to bottom, show representative images of Virchow's space, corpus callosum, IVth ventricle, and cortical necrosis observed in one mouse from n = 3 mice studied (likely caused by cortical ischaemia at time of death). In two mice mild expansion of the Virchow's space was observed, that was likely caused by a fixation/processing artefact rather than oedema because proteinaceous fluid is absent. The scale bars are: 4x magnification, 50μm; 10x magnification, 120μm; 20x magnification, 300μm.

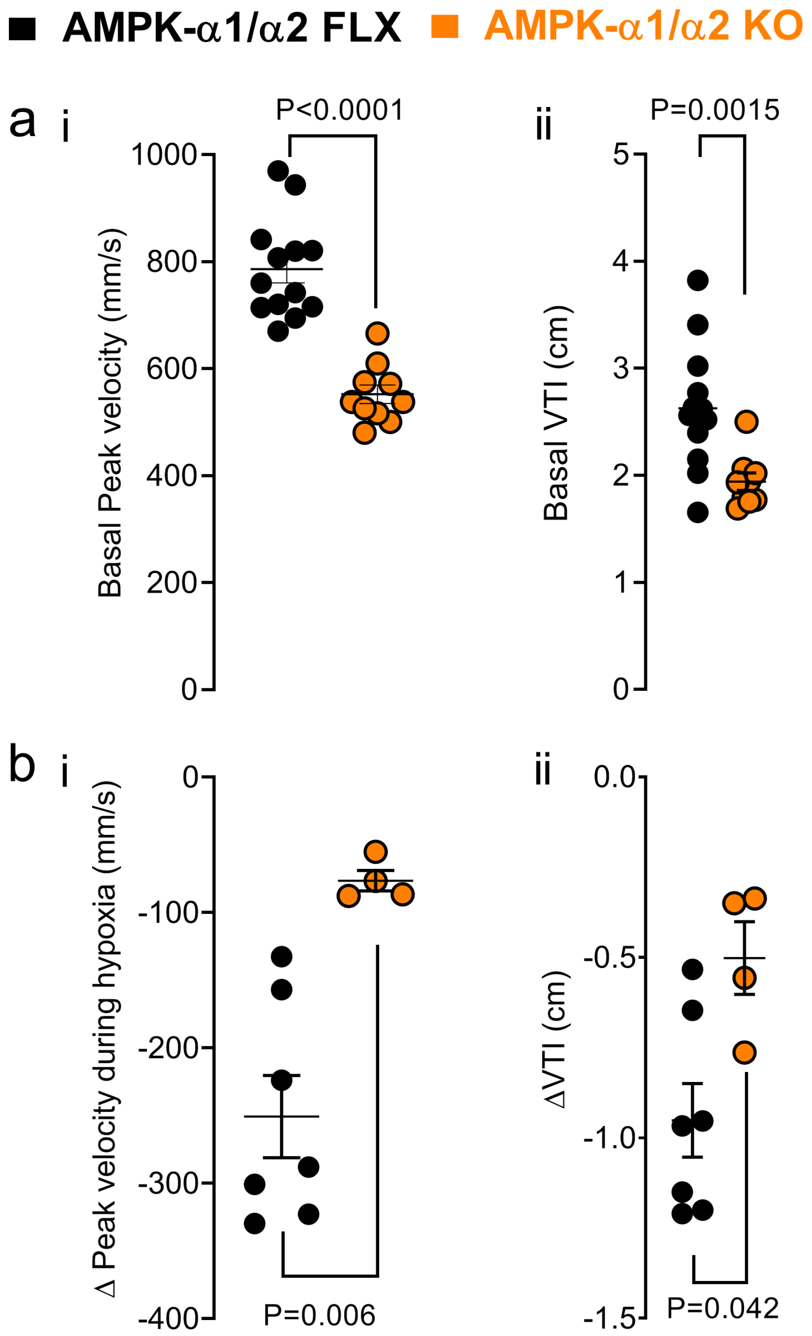

Supplementary Figure 10. Reduced normoxic pulmonary flow and impaired hypoxic pulmonary vasoconstriction in AMPK-α1/α2 identified by parallel changes in peak velocity and velocity time integral. a, Scatter plots show normoxic (i) peak velocity and (ii) normoxic velocity time integral (VTI) for AMPK-α1/α2 floxed (AMPK-α1/α2 FLX, n = 13 mice) and AMPK-α1/α2 knockouts (AMPK-α1/α2 KO, n = 10 mice). b, Scatter plots for maximum change in (i) peak velocity and (ii) VTI observed during exposure of AMPK-α1/α2 FLX (n = 7 mice) and AMPK-α1/α2 KO (n = 4 mice) mice to 8% O_2. Data are expressed as mean ± SEM. Statistical significance was assessed by two- sided unpaired Student's t test (ai) and two- sided unpaired Mann-Whitney's test (aii, bi-ii).

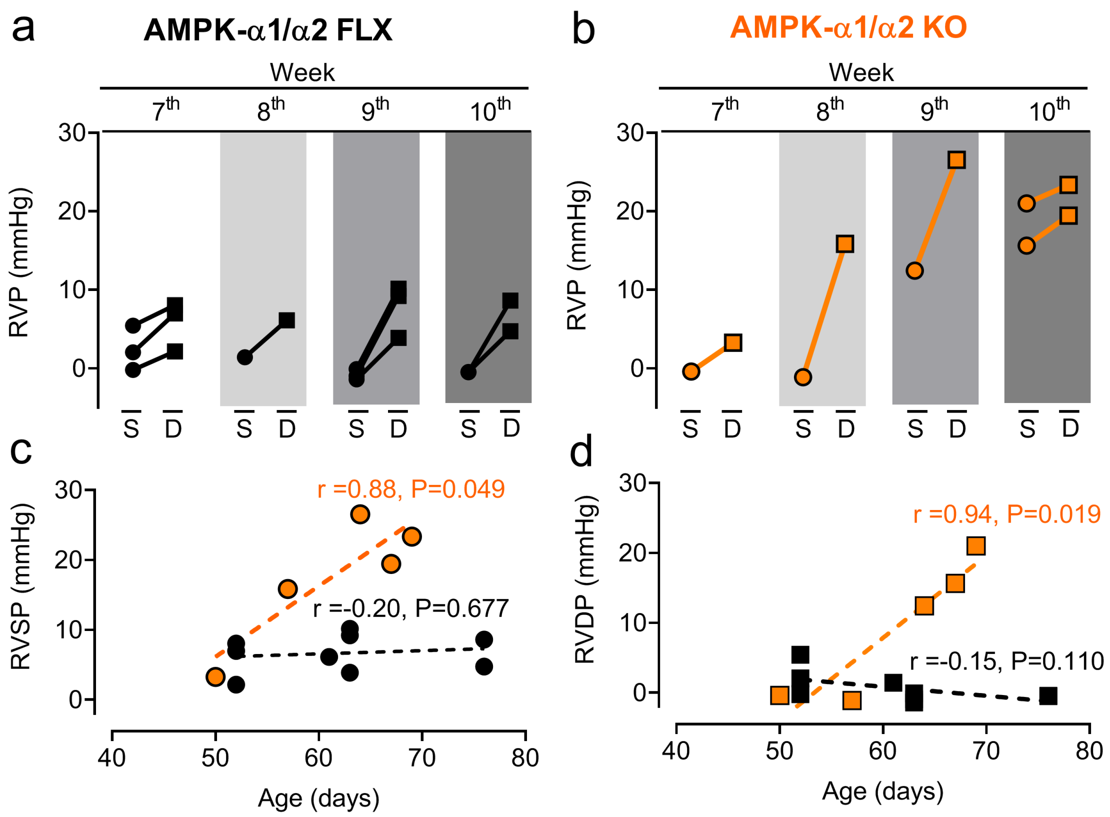

Supplementary Figure 11. Right ventricular systolic pressure of AMPK-α1/α2 knockouts increases rapidly with age. Graphs illustrate age-dependent changes in right ventricular systolic (S) and diastolic (D) pressures for (a) AMPK-α1/α2 floxed (AMPK-α1/α2 FLX, n = 9) and (b) AMPK-α1/α2 knockout (AMPK-α1/α2 KO, n = 5) mice. Scatter plots show values of (c) right ventricular systolic and (d) diastolic pressures against age for AMPK-α1/α2 FLX and AMPK-α1/α2 KO mice. Linear regression analysis indicates (coefficient of determination r^2 = 0.77) that right ventricular systolic pressure of AMPK-α1/α2 KO increases rapidly with age between 50 and 70 days (orange), while for age-matched AMPK-α1/α2 FLX there was no correlation between right ventricular systolic pressure and age (black). Statistical significance was assessed by two- sided Pearson correlation coefficients.

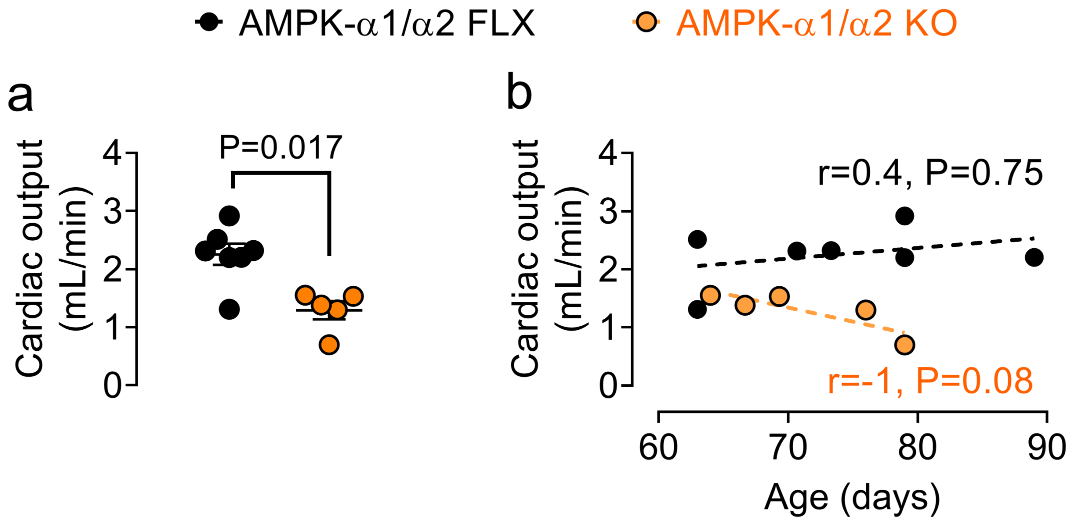

Supplementary Figure 12. Right ventricular cardiac output of AMPK-α1/α2 knockouts decreases with age. Scatter plots show (a) mean ± SEM for right ventricular cardiac output and (b) age-dependent changes in right ventricular cardiac output for AMPK-α1/α2 floxed (AMPK-α1/α2 FLX; n = 7) and AMPK-α1/α2 knockout (AMPK-α1/α2 KO; n = 5) mice. Linear regression analysis indicates (coefficient of determination R^2 = -0.75) that right ventricular cardiac output of AMPK-α1/α2 KO decreases with age between 50 and 70 days (orange), while for age-matched AMPK-α1/α2 FLX there was no correlation between cardiac output and age between 50 and 70 days (black). Cardiac output was derived from the heart rate and flow volume calculated by the velocity-time integral (VTI) and cross-sectional area (CSA) of the valve: CO [L/min] = HR x VTI x CSA (cross sectional area of murine pulmonary artery; diameter = 0.44 ± 0.06[1]; $A=\pi r^2$). Statistical significance was assessed by two-sided unpaired Mann-Whitney's test (a) and two-sided Pearson correlation coefficients (b).

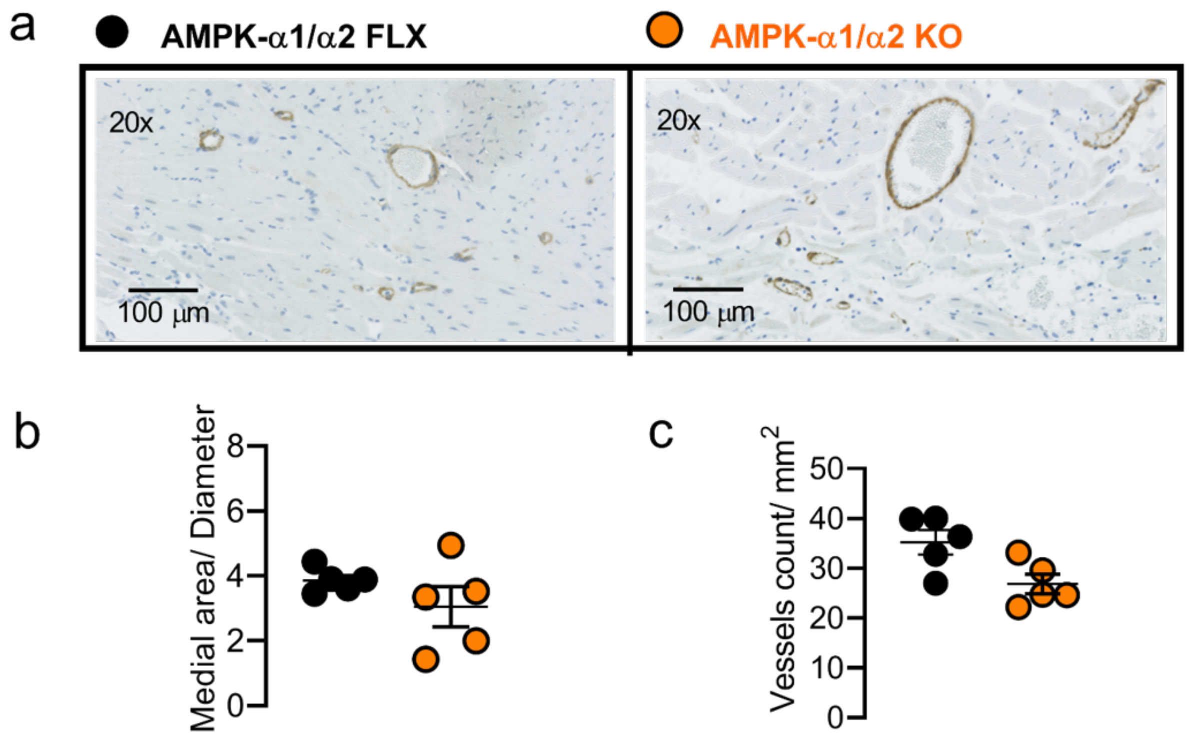

Supplementary Figure 13. AMPK-α1/α2 deletion driven by Cre expression via the transgelin promoter does not affect right ventricular blood vessel number or medial thickness. a, Representative images of right ventricles from samples stained for α-smooth muscle actin. Scatter plot shows measurements of (b) medial area/diameter (n = 5 mice per genotype, average of 20-24 arteries per mouse, n = 4 fields per mouse) and (c) vessel count/mm^2 from AMPK-α1/α2 KOs (n= 5 mice, average of 3-4 mm^2 area per mouse) vs age-matched AMPK-α1/α2 floxed controls (n=5 mice, average of 3-4 mm^2 area per mouse). Data are expressed as mean± SEM. Statistical significance was assessed by two- sided unpaired Mann-Whitney's test.

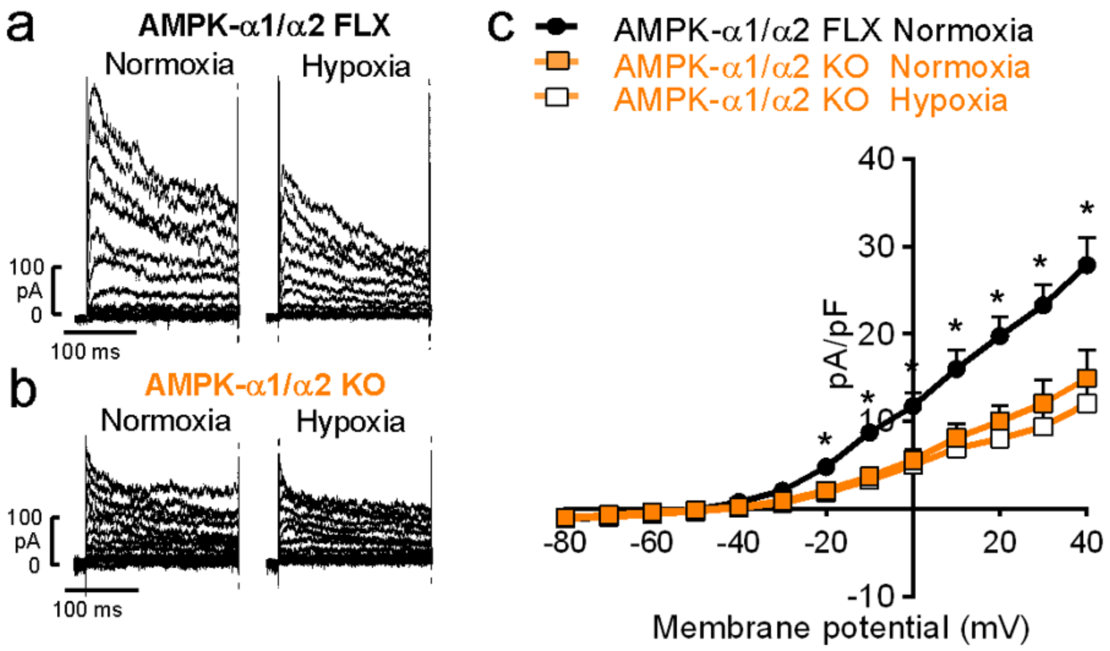

Supplementary Figure 14. AMPK-α1/α2 deletion reduces K_V1.5 current density and blocks K_V inhibition by hypoxia. a-b, Panels show example records for K_V currents recorded during control conditions and following extracellular during hypoxia (~6% O_2) in acutely isolated pulmonary arterial myocytes from either (a) AMPK-α1/α2 floxed (AMPK-α1/α2 FLX) or (b) AMPK-α1/α2 knockouts (AMPK-α1/α2 KO). c, Comparison of current-voltage relationship for K_V currents recorded in pulmonary arterial myocytes from AMPK-α1/α2 floxed (n = 3 cells from n = 3 mice) and AMPK-α1/α2 KO (n = 4 cells from n = 3 mice) under control conditions, and the effect of hypoxia the current-voltage relationship of K_V currents in pulmonary arterial myocytes from AMPK-α1/α2 KOs. Data are expressed as mean± SEM. Statistical significance was assessed by two- sided paired (normoxia vs hypoxia) and two- sided unpaired Student's t test (AMPK-α1/α2 FLX vs AMPK-α1/α2 KO). P values in c for AMPK-α1/α2 FLX vs AMPK-α1/α2 KO: -20mV, * = 0.0305; -10mV, * = 0.0194; 0mV, * = 0.0214; 10mV, * = 0.0295; 20mV, * = 0.0195; 30mV, * = 0.0318; 40mV, * = 0.0379.

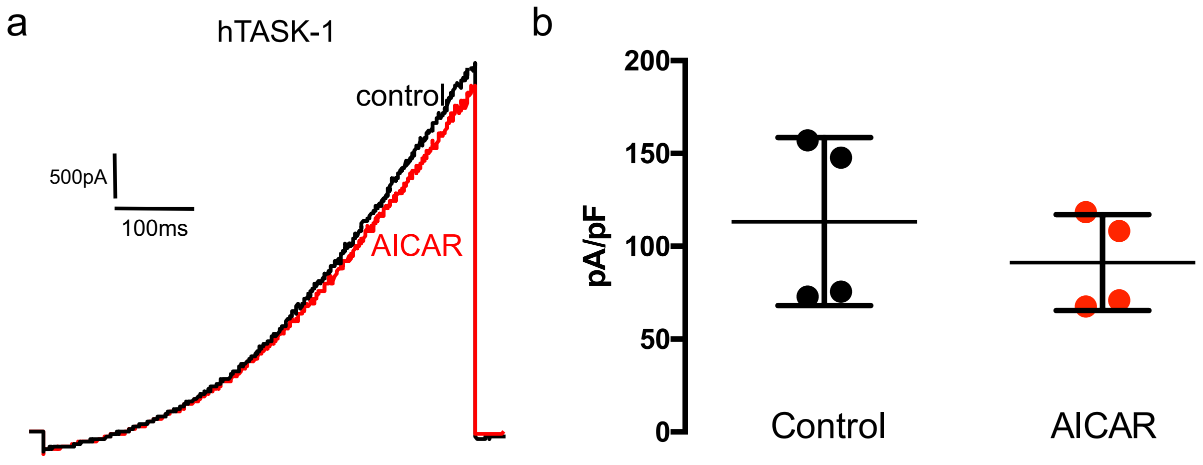

Supplementary Figure 15. AMPK activation does not regulate TASK-1 potassium currents. a, shows example records for K^+ currents in HEK293 cells transfected with human KCNK3 (hTASK-1) under control conditions and after pre-incubation (1h) with AICAR (1mM). b, shows dot plots of mean ± SEM for the peak hTASK-1 current recorded at +100mV under control conditions (n = 4 cells) with that recorded in the presence of AICAR (1mM; n = 4 cells). Data are expressed as mean± SEM. Statistical significance was assessed by two-sided unpaired Student's t test.

Right ventricle

	AMPK-α1/α2 FLX			AMPK-α1 KO			AMPK-α2 KO			AMPK-α1/α2 KO		
	Mean	SEM	n	Mean	SEM	n	Mean	SEM	n	Mean	SEM	n
Systolic Volume (μL)	2.2	± 0.9	4	1.3	± 0	4	2.6	± 0.9	4	7.4	± 1.5	5 (P=0.0264)
Diastolic Volume (μL)	9	± 2.6	4	6.5	± 0.7	4	7.1	± 1.1	4	12.4	± 2.4	5
Stroke Volume (μL)	6.8	± 1.8	4	5.2	± 0.6	4	4.5	± 0.4	4	5.1	± 1.9	5
Ejection Fraction (%)	78.4	± 4.4	4	79.4	± 1.6	4	66.4	± 7.8	4	38.3	± 10.6	5 (P=0.0065)
Fractional Shortering (%)	44.7	± 4.3	4	44.8	± 1.7	4	35	± 5.8	4	17.9	± 5.5	5
Cardiac Output (mL/min)	3.7	± 0.9	4	2.9	± 0.3	4	2.4	± 0.3	4	2.4	± 0.9	5

Left ventricle

	AMPK-α1/α2 FLX			AMPK-α1 KO			AMPK-α2 KO			AMPK-α1/α2 KO		
	Mean	SEM	n	Mean	SEM	n	Mean	SEM	n	Mean	SEM	n
Systolic Volume (μL)	20.8	± 3.1	4	16.2	± 4	4	22.6	± 4.3	4	105.7	± 17.4	5 (P=0.0004)
Diastolic Volume (μL)	72.7	± 3.7	4	61.2	± 7.7	4	62.8	± 5.1	4	129.8	± 17.6	5 (P=0.0111)
Stroke Volume (μL)	51.9	± 1.8	4	45.1	± 6.4	4	40.2	± 1.1	4 (P=0.0286)	24.2	± 3.2	5 (P=0.0030)
Ejection Fraction (%)	71.8	± 3.1	4	73.9	± 5.2	4	65	± 4.1	4	20	± 3.7	5 (P=0.0184)
Fractional Shortering (%)	40.8	± 2.6	4	42.6	± 4.4	4	35.3	± 3.1	4	9.2	± 1.8	5 (P=0.0184)
Cardiac Output (ml/min)	28.5	± 0.9	4	25.4	± 2.9	4	21.1	± 0.3	4 (P=0.0286)	11.8	± 1.9	5 (P=0.0030)
Heart rate (bpm)	569.1	± 9.1	4	576.7	± 8.5	4	541.2	± 13.4	4	508.4	± 10.2	5 (P=0.0004)

Pulmonary flow

	AMPK-α1/α2 FLX			AMPK-α1 KO			AMPK-α2 KO			AMPK-α1/α2 KO		
	Mean	SEM	n	Mean	SEM	n	Mean	SEM	n	Mean	SEM	n
Peak velocity (mm/s)	785.9	± 25.7	13	737.5	± 38.3	5	721.7	± 20.2	7	552	± 17.4	10 (P=0.0279)
VTI (cm)	2.7	± 0.3	13	2.5	± 0.1	5	2.7	± 0.1	7	1.7	± 0.1	10 (P=0.0383)

Supplementary Table 1. AMPK-α1/α2 deletion causes right and left ventricular myopathy and dysfunction. Doppler ultrasound parameters for right and left ventricular function for AMPK-α1/α2 floxed (AMPK-α1/α2 FLX), AMPK-α1 knockouts (AMPK-α1 KO), AMPK-α2 knockouts (AMPK-α2 KO) and AMPK-α1/α2 knockouts (AMPK-α1/α2 KO). Data are expressed as mean± SEM. Statistical significance was assessed by Kruskall Wallis test with Dunn’s correction for multiple comparisons.

AMPK-α1/α2 KO	Lateral ventricles	Corpus callosum/ Striatum/ Cortex area	Cortical neuronal necrosis	Enlargement of Virchow's space
**1**	Normal	Normal	No	Mild
**2**	Normal	Normal	Yes	No
**3**	Normal	Normal	No	Mild

Supplementary Table 2. Qualitative histological assessment of brain sections of terminal AMPK-α1/α2 knockouts reveals no central pathology. Qualitative histological assessment (exemplar images in Supplementary Figure 9) of surface area identifies no evidence of ventricular expansion or reduction of striatal, cortical and corpus callosum volumes in section of brains from n =3 AMPK-α1/α2 knockouts. In two of the mice mild expansion of the Virchow's space was observed, that is likely caused by a fixation/processing artefact rather than edema because proteinaceous fluid is absent. One mouse exhibited signs of cortical neuronal necrosis, most likely caused by cortical ischaemia secondary to cardiac failure.

References

1. James IA, Yi T, Tara S, Best CA, Stuber AJ, Shah KV, Austin BF, Sugiura T, Lee YU, Lincoln J, Trask AJ, Shinoka T and Breuer CK. Hemodynamic Characterization of a Mouse Model for Investigating the Cellular and Molecular Mechanisms of Neotissue Formation in Tissue-Engineered Heart Valves. *Tissue Eng Part C Methods*. 2015;21:987-94.
