# Supplementary material for: AMPK deficiency in smooth muscles causes persistent pulmonary hypertension of the new-born and premature death
Source: Nat Commun. 2022 Aug 26;13:5034. doi: 10.1038/s41467-022-32568-7 (PMC9418192; doi:10.1038/s41467-022-32568-7)
Supplement: Supplementary file 2 — Reporting Summary [file 41467_2022_32568_MOESM2_ESM.pdf]

nature portfolio

Corresponding author(s): A. MArk Evans

Last updated by author(s): 04/07/2022

Reporting Summary

Nature Portfolio wishes to improve the reproducibility of the work that we publish. This form provides structure for consistency and transparency in reporting. For further information on Nature Portfolio policies, see our Editorial Policies and the Editorial Policy Checklist.

Statistics

For all statistical analyses, confirm that the following items are present in the figure legend, table legend, main text, or Methods section.

n/a	Confirmed	
☐	☒	The exact sample size (*n*) for each experimental group/condition, given as a discrete number and unit of measurement
☐	☒	A statement on whether measurements were taken from distinct samples or whether the same sample was measured repeatedly
☐	☒	The statistical test(s) used AND whether they are one- or two-sided<br>*Only common tests should be described solely by name; describe more complex techniques in the Methods section.*
☐	☒	A description of all covariates tested
☐	☒	A description of any assumptions or corrections, such as tests of normality and adjustment for multiple comparisons
☐	☒	A full description of the statistical parameters including central tendency (e.g. means) or other basic estimates (e.g. regression coefficient) AND variation (e.g. standard deviation) or associated estimates of uncertainty (e.g. confidence intervals)
☐	☒	For null hypothesis testing, the test statistic (e.g. *F*, *t*, *r*) with confidence intervals, effect sizes, degrees of freedom and *P* value noted<br>*Give P values as exact values whenever suitable.*
☒	☐	For Bayesian analysis, information on the choice of priors and Markov chain Monte Carlo settings
☒	☐	For hierarchical and complex designs, identification of the appropriate level for tests and full reporting of outcomes
☒	☐	Estimates of effect sizes (e.g. Cohen's *d*, Pearson's *r*), indicating how they were calculated

Our web collection on statistics for biologists contains articles on many of the points above.

Software and code

Policy information about availability of computer code

Data collection	Volocity 5.5.1 image acquisition and analysis software, Clampex 10, Vevo770 (Visualsonics Inc), QuPath (v0.3.2; https://qupath.github.io/), FIJI (GNU General Public Licence, built on ImageJ), NDP view 2 software (Hamamatsu Photonics Ltd, U.K.).
Data analysis	Volocity 5.5.1 image acquisition and analysis software, Clampex 10, Clampfit 10, Graphpad Prism 6, ImageJ (Ver 1.41, NIH, Bethesda, MD, USA), NDP view 2 software (Hamamatsu Photonics Ltd, U.K.), Vevo770 (Visualsonics Inc), QuPath (v0.3.2; https://qupath.github.io/), FIJI (GNU General Public Licence, built on ImageJ)

For manuscripts utilizing custom algorithms or software that are central to the research but not yet described in published literature, software must be made available to editors and reviewers. We strongly encourage code deposition in a community repository (e.g. GitHub). See the Nature Portfolio guidelines for submitting code & software for further information.

Data

Policy information about availability of data

All manuscripts must include a data availability statement. This statement should provide the following information, where applicable:

- Accession codes, unique identifiers, or web links for publicly available datasets
- A description of any restrictions on data availability
- For clinical datasets or third party data, please ensure that the statement adheres to our policy

All data points are presented in the manuscript and/or supplementary information. Any further information required will be made available by the corresponding author upon request

Human research participants

Policy information about studies involving human research participants and Sex and Gender in Research.

Reporting on sex and gender	NA
Population characteristics	NA
Recruitment	NA
Ethics oversight	NA

Note that full information on the approval of the study protocol must also be provided in the manuscript.

Field-specific reporting

Please select the one below that is the best fit for your research. If you are not sure, read the appropriate sections before making your selection.

[x] Life sciences [ ] Behavioural & social sciences [ ] Ecological, evolutionary & environmental sciences

For a reference copy of the document with all sections, see nature.com/documents/nr-reporting-summary-flat.pdf

Life sciences study design

All studies must disclose on these points even when the disclosure is negative.

Sample size	To ensure that outcomes are robust to inter-animal variability, group data typically show a standard deviation of ~10% of the mean. For an experiment with a single control group, a group size of 8 yields ~80% power to detect between group differences of >15% and is therefore generally large enough to be meaningful.<br>However, all experiments were conducted in stages. In doing so, the variable criteria sequential stopping rule (SSR) was employed to ensure as few mice as possible are used to determine by significance test when a given experiment is stopped or continued, without increasing type I errors. For example, three (3) mice at a time can be used with repeat tests for significance until: (A) a significant effect is detected; (B) no effect is seen, or significance is not reached. In this way animal use will be minimised, power will be maximised, and the probability of type I errors will be kept constant. SSR can require 30% fewer mice to achieve similar power to that predicted by a Power Calculation. SSR provides a formal method for not only minimising but estimating animal use whether one employs a t test, one way ANOVA, or even, in some circumstances, multifactorial ANOVA.<br>Where statistical analysis was not employed (e.g., Western blot gels, qRT-PCR gels) samples from at least three different animals were tested.
Data exclusions	Electrophysiological data were excluded if a greater than 20% increase in series resistance occurred during the recording. Only cells with a seal resistance was >3Gohms were accepted for analysis. No other data were exlcuded.
Replication	All outcomes were highly reproducible and were robust to inter-animal variability. All experiments were conducted in stages. In doing so, the variable criteria sequential stopping rule (SSR) was employed to ensure as few mice as possible are used to determine by significance test when a given experiment is stopped or continued, without increasing type I errors. For example, three (3) mice at a time can be used with repeat tests for significance until: (A) a significant effect is detected; (B) no effect is seen, or significance is not reached. In this way animal use will be minimised, power will be maximised, and the probability of type I errors will be kept constant. SSR can require 30% fewer mice to achieve similar power to that predicted by a Power Calculation. SSR provides a formal method for not only minimising but estimating animal use whether one employs a t test, one way ANOVA, or even, in some circumstances, multifactorial ANOVA.<br>Where statistical analysis was not employed (e.g., Western blot gels, qRT-PCR gels) samples from at least three different animals were tested.
Randomization	Paired (control + knockout) were routinely used for all experiments. Animals from multiple litters were assessed over a five year period, except for neonates which were from three different litters and assessed over two months.

Blinding	All histology was completed and analysed blind. By their nature ultrasound, electrophysiology and mitochondrial imaging could not be completed blind. However, data analyses were completed and cross-checked blind.

Reporting for specific materials, systems and methods

We require information from authors about some types of materials, experimental systems and methods used in many studies. Here, indicate whether each material, system or method listed is relevant to your study. If you are not sure if a list item applies to your research, read the appropriate section before selecting a response.

Materials & experimental systems

n/a	Involved in the study
☐	☒ Antibodies
☐	☒ Eukaryotic cell lines
☒	☐ Palaeontology and archaeology
☐	☒ Animals and other organisms
☒	☐ Clinical data
☒	☐ Dual use research of concern

Methods

n/a	Involved in the study
☒	☐ ChIP-seq
☒	☐ Flow cytometry
☒	☐ MRI-based neuroimaging

Antibodies

Antibodies used	Anti-smooth muscle α-actin antibody (ab5694, Abcam, UK; 1:400 dilution).<br>Anti-Ki67 antibody (1:400 dilution; clone MiB1400, Agilent-Dako).<br>Anti-AMPK-α1 antibody (ab3759, cell signalling, 1:1000 dilution, Abcam).<br>Anti-AMPK-α2 antibody (1:1000 dilution; ab105028, cell signalling, Abcam).<br>Anti-GAPDH antibody (1:10,000 dilution; 2118S, cell signalling, Abcam).
Validation	AMPK-α1 and 2 antibodies were validated against AMPK positive and null fibroblasts and HEK293 cells. antibody. For manufacturer's validation of Anti-AMPK-α1 and references see:https://www.abcam.com/ampk-alpha-1-antibody-ab3759.html. For Anti-AMPK-α2 validation and references see: https://www.abcam.com/ampk-alpha-2-antibody-ab105028.html<br>Anti-α-smooth muscle actin antibody and shown by us to selectively label smooth muscles. For manufacturer's validation and references see: https://www.abcam.com/products?keywords=Anti-smooth+muscle+%CE%B1-actin+antibody+ab5694.<br>Anti-Ki67 antibody. For manufacturer's validation and references see:<br>https://www.agilent.com/en/product/immunohistochemistry/antibodies-controls/primary-antibodies/ki-67-antigen-(concentrate)-76646<br>Anti-GAPDH GAPDH antibody selectively labeled protein bands of of the expected molecular weight by Western blot. For manufacturer's validation and references see: cellsignal.com/products/primary-antibodies/gapdh-14c10-rabbit-mab/2118?site-search-type=Products&N=4294956287&Ntt=gapdh+2118s+&fromPage=plp&_requestid=260267

Eukaryotic cell lines

Policy information about cell lines and Sex and Gender in Research

Cell line source(s)	HEK293 cells stably expressing hTASK-1 were were developed from in-house stocks of HEK293 cells that have been maintained for >15 years. Originally sourced from Cambridge Biosciences.
Authentication	To generate HEK293 cell lines stably expressing wild-type hTASK-1, cells were transfected with either pRAT/hTASK-1 constructs using the PolyFect transfection reagent (Qiagen, Hybaid Ltd, Teddington, UK) according to manufacturer’s instructions. Stable HEK293 cell lines were achieved by antibiotic selection with G-418 (1mg/ml, Gibco-BRL, Paisley, UK) added to the medium 3 days after transfection. Selection was applied for 4 weeks (media changed every 4-5 days), after which time individual colonies were picked and seeded in T25 flasks and allowed to reach confluence.
Mycoplasma contamination	All cells tested were free of Mycoplasma contamination
Commonly misidentified lines (See ICLAC register)	*Name any commonly misidentified cell lines used in the study and provide a rationale for their use.*

Animals and other research organisms

Policy information about studies involving animals; ARRIVE guidelines recommended for reporting animal research, and Sex and Gender in Research

Laboratory animals	AMPK-α1 and AMPK-αs floxed mice were obtained from Benoit Viollet. Trangelin-Cre mice were from Jax Labs. All mice were bfred on a C57/BL6 background.
Wild animals	NA

Reporting on sex	Males and females were used as reported. No gender differences were identified in the AMPK knockout mice
Field-collected samples	NA
Ethics oversight	All experiments were performed in accordance with the regulations of the United Kingdom Animals (Scientific Procedures) Act of 1986. All studies and breeding were approved by the University of Edinburgh, College of Medicine Animal Welfare and Ethics Review Committee and performed under UK Home Office project license held by AME (PBA4DCF9D). Both male and female mice were used, all of which were on a C57/Bl6 background. Numbers of mice (≥ 3 per measure) used are indicated for each experiment.

Note that full information on the approval of the study protocol must also be provided in the manuscript.
